# MinYS: mine your symbiont by targeted genome assembly in symbiotic communities

**DOI:** 10.1093/nargab/lqaa047

**Published:** 2020-07-03

**Authors:** Cervin Guyomar, Wesley Delage, Fabrice Legeai, Christophe Mougel, Jean-Christophe Simon, Claire Lemaitre

**Affiliations:** Univ. Rennes, Inria, CNRS, IRISA, F-35000 Rennes, France; Institut de Génétique, Environnement et Protection des Plantes (IGEPP), INRAE, Institut Agro, Univ. Rennes, F-35653 Le Rheu, France; iDiv—German Centre for Integrative Biodiversity Research, Deutscher Platz 5e, D-04103 Leipzig, Germany; Univ. Rennes, Inria, CNRS, IRISA, F-35000 Rennes, France; Institut de Génétique, Environnement et Protection des Plantes (IGEPP), INRAE, Institut Agro, Univ. Rennes, F-35653 Le Rheu, France; Institut de Génétique, Environnement et Protection des Plantes (IGEPP), INRAE, Institut Agro, Univ. Rennes, F-35653 Le Rheu, France; Institut de Génétique, Environnement et Protection des Plantes (IGEPP), INRAE, Institut Agro, Univ. Rennes, F-35653 Le Rheu, France; Univ. Rennes, Inria, CNRS, IRISA, F-35000 Rennes, France

## Abstract

Most metazoans are associated with symbionts. Characterizing the effect of a particular symbiont often requires getting access to its genome, which is usually done by sequencing the whole community. We present MinYS, a targeted assembly approach to assemble a particular genome of interest from such metagenomic data. First, taking advantage of a reference genome, a subset of the reads is assembled into a set of backbone contigs. Then, this draft assembly is completed using the whole metagenomic readset in a *de novo* manner. The resulting assembly is output as a genome graph, enabling different strains with potential structural variants coexisting in the sample to be distinguished. MinYS was applied to 50 pea aphid resequencing samples, with variable diversity in symbiont communities, in order to recover the genome sequence of its obligatory bacterial symbiont, *Buchnera aphidicola*. It was able to return high-quality assemblies (one contig assembly in 90% of the samples), even when using increasingly distant reference genomes, and to retrieve large structural variations in the samples. Because of its targeted essence, it outperformed standard metagenomic assemblers in terms of both time and assembly quality.

## INTRODUCTION

Advances of molecular techniques have greatly contributed to the recognition of the importance of microorganisms in every ecosystem. In particular, it is now well established that most metazoans are associated with microbes, forming complex entities referred to as holobionts. In these systems, microbes are interacting together as well as with their host, and host–microbe interactions can have significant effects on the host phenotype (1). Many animals have microbial associations with one or a few specific symbionts. This is, for example, the case for corals associated with the algal symbiont *Symbiodinium* (2), squids with *Vibrio* (3), woodlice with *Wolbachia* (4) or hemipteran insects with specific obligatory and facultative bacterial symbionts (5). As symbionts are generally not cultivable outside the host, the whole community is usually sequenced, resulting in a metagenomic dataset mixing host and symbiont reads. These datasets are unbalanced: the great majority of the reads often originate from the host genome, but since the genomes of the symbionts are often several orders of magnitude smaller than that of the eukaryotic host, symbiont genomes can have large read depth in such samples. This enables the extraction of relevant information about the symbionts, but requires significant effort, since the host reads are a computational burden for most analyses. In this context, providing bioinformatic tools that enable the assembly of a particular genome of interest from a metagenomic sample, ignoring the overwhelming amount of reads from other organisms, would greatly accelerate the characterization of symbiont genomes, and therefore decipher particular host–symbiont relationships.

In many cases, some knowledge and genomic resources about the symbiont of interest are already available. A common problem is to recover the full genomic sequence of the particular symbiont present in the metagenomic dataset, using available genomic resources. The way we address this problem depends on the availability of a reference genome and its closeness to the considered species. In the easiest but rarest case, a reference genome of good quality is available for the considered species, and a mapping-based approach is classically performed. Reads from the whole metagenomic datasets are mapped on this reference genome and small variants are called to characterize the strain at play. This approach does not output *per se* a genome sequence but rather a list of punctual variants with respect to a reference genome, without any evidence that this list is exhaustive and ignoring potential structural differences, such as large novel insertions or deletions. This approach may therefore miss crucial genomic information even when a very close reference genome is available, since it is well known that bacterial strains can have highly variable accessory genomes, potentially responsible for pathogenicity or other phenotypic effects (6).

To circumvent these drawbacks, a more classical approach relies on *de novo* genome assembly to obtain full genomic sequences. In this metagenomic context, the whole community is assembled and available genomic resources are used afterward to select among the assembled contigs the ones originating from the species of interest. Contig binning tools have been designed specifically for the separation of host and symbiont contigs, such as BlobTools (7) and Autometa (8). They are based on different signals computed on the contigs, including similarity with available genomic resources, but also nucleotide composition and read depth. However, their performances depend on the quality of the initial whole metagenomic assembly. Genome assembly from metagenomic data is a challenging task, because of both the many species coexisting in the samples and the polymorphism within these species. Although many recent software are devoted to this task (9–11), metagenomic assemblies are often very fragmented and come with a high computational cost (12). In the particular case where reads originating from the targeted organism are a minority in the whole metagenomic readset, this computational cost could be reduced. In addition, one would expect that focusing on a subsample of reads would lead to less fragmented assemblies.

Therefore, combining mapping-based and assembly-based approaches in a so-called targeted genome assembly seems a promising way to facilitate the characterization of specific symbionts in metagenomic datasets. In such approaches, a subset of the metagenomic reads is first recruited by mapping to a reference genome and then *de novo* assembled. The relative proximity between the targeted and the reference genomes is a key parameter of the approach, and the methods must be able to incorporate non-recruited reads in the assembly, in order not to miss divergent or strain-specific regions.

Several tools, such as MITObim (13), NOVOPlasty (14), LOCAS (15), Pilon (16) or IMR/DENOM (17), were designed following the idea of combining mapping to a reference and *de novo* assembly. However, they all have limitations that make them unsuitable to handle metagenomic data. Primarily, except for NOVOPlasty, these methods rely on the assumption of genomic homogeneity (no polymorphism), which is very rarely met in a metagenomic context. Therefore, they are not suited to detect and characterize coexisting strains in metagenomic datasets. Moreover, these methods generally use the architecture of the reference genome as a starting point for the assembly, and are therefore unable to deal with large structural variants that differ from the reference genome (IMR/DENOM, Pilon, LOCAS). Finally, some tools are designed either for short organelle genomes (MITObim, NOVOPlasty) or for small resequencing datasets (LOCAS) and they are not scaling up to the size of metagenomic datasets.

To our knowledge, a single tool has been designed to guide the assembly by a reference genome in a metagenomic context. MetaCompass is a pipeline described in a pre-print (18) that (i) recruits a subset of reads by mapping on a reference genome, (ii) assembles them into contigs and (iii) assembles all the remaining non-recruited reads to recover the regions potentially missing in the reference genome. This last step ultimately amounts to assemble the whole community instead of one single genome of interest, making it a reference-guided rather than a targeted approach.

In this work, we present MinYS, a novel method for the assembly of a microbial genome of interest from metagenomic data that does not require assembling the full readset. This method can recover large regions absent from the reference genome, makes no assumption about the order and orientation of the regions homologous to the reference and is capable of returning several different solutions associated with the strain diversity within the sample. It is based on two main steps, a reference-based recruitment and assembly of reads, followed by a targeted assembly using the whole readset, filling in the gaps between the previously assembled contigs.

We applied this method to reconstruct genomes from 50 metagenomic samples of the pea aphid *Acyrthosiphon pisum*. Focusing on this aphid’s primary endosymbiont, *Buchnera aphidicola*, we demonstrated the ability of MinYS to assemble complete bacterial genomes in a single contig using a remote reference genome as a primer.

## MATERIALS AND METHODS

### A novel method for targeted genome assembly with metagenomic data

#### Approach overview

**Figure 1.**
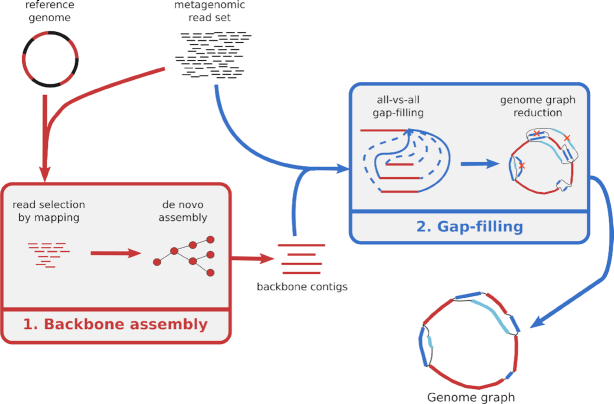
Overview of MinYS approach, for targeted genome assembly. MinYS takes as input a reference genome and a set of metagenomic reads, and outputs the targeted genome assembly as a genome graph encompassing the potential strain diversity contained in the sample.

The method described in this work relies on a two-step pipeline, described in Figure 1.

The first step uses a given reference genome to build an incomplete but trustworthy assembly from a subset of the input metagenomic reads. The result of this step is hereafter referred to as the backbone contigs. The second step uses the whole set of metagenomic reads to extend the previously assembled contigs and to form a complete assembly, without *a priori* on the order and orientation of the backbone contigs. The result of the pipeline is a genome graph encompassing the structural diversity detected among the assembled genome. This graph can be exploited by extracting contigs, or paths of the graph that represent different strains of the targeted genome.

#### Assembly of backbone contigs

The first step requires a metagenomic readset and a reference genome, and returns contigs that are *de novo* assembled using only reads mapped on the reference genome. All metagenomic reads are mapped against the reference genome using BWA MEM (19), and the mapped reads are kept and *de novo* assembled using the Minia (20) assembler. Although any assembler can be used in this step, we used Minia for its low memory footprint, and its assembly algorithm similar to the one used in the second step of the method. The amount of mapped reads and the size and contiguity of the resulting assembly depend on the sequence similarity between the reference genome and the targeted genome. However, the goal of this step is not to produce a complete assembly of the targeted genome but rather to generate high-quality contigs that can reliably be used for the upcoming gap-filling step. To achieve this, we set up Minia with more stringent parameters than for a usual assembly task (i.e. with a higher *k*-mer size and a higher minimal abundance threshold). For the same reasons, only contigs larger than a user-defined threshold (400 bp by default) were kept for the second step.

#### Gap filling between backbone contigs

The essential step of the pipeline is the gap filling between backbone contigs, which enables the assembly of regions of the targeted genome that are absent or too much divergent in the reference genome. This is made possible by a targeted assembly of the whole readset using the backbone contigs as primers. This step does not require any relative ordering or orientation of contigs, since all possible combinations are tested during gap filling. As a result, structural variants can be detected, either with respect to the reference genome or within the sample.

This step is based on a module of the software MindTheGap, originally developed for the detection and assembly of long insertion variants (21). MindTheGap is built upon the GATB library (22), which offers memory- and time-efficient data structures for de Bruijn graphs. The ‘fill’ module of MindTheGap builds a de Bruijn graph of the entire input readset, and performs a local assembly between the left and the right *k*-mers of each insertion site, by looking for all the paths in the de Bruijn graph starting from the left (source) *k*-mer and ending in the right (target) *k*-mer. In this work, we took advantage of this module of MindTheGap and adapted it to the problem of simultaneous gap filling between multiple contigs. It has been modified to make possible the gap filling between a source *k*-mer and multiple target *k*-mers, enabling the ‘all versus all’ gap filling within a set of contigs with a linear time increase. The improvements that have been made to MindTheGap in the context of MinYS are available as a new option of MindTheGap (named contig mode) and are presented in Section S1 and Supplementary Figure S1 in the Supplementary Data.

MindTheGap gap-filling results are output as a sequence graph in the GFA (graphical fragment assembly) format, containing all input contigs and their successful gap-filling sequences as nodes, together with their overlap relationships as edges.

#### Graph simplification

By construction, the raw sequence graph output by MindTheGap is likely to contain redundant sequence information. A graph simplification step has been therefore developed to remove uninformative sequence redundancy and hence ease the analysis and usage of such a genome graph. The different steps of this process are represented in Supplementary Figure S2.

First, it is likely that two contigs are linked in the graph by two gap-fillings with reverse-complement sequences, one starting from the left contig and the other one starting from the right contig in the reverse orientation. Such reciprocal links are removed when their sequence identity is over a 95% identity threshold.

Second, when several gap-filling sequences start (or end) from the same source contig, it is possible that a subset of them has an identical prefix (suffix) and starts to diverge only after a potential large distance from the source (target) contig. This results in redundant sequence information in the final sequence set. A node merging algorithm is applied in order to return a final set of sequences (nodes of the graph) that do not share large identical subsequences as prefix or suffix. Sets of sequences sharing the same prefix of size *l* are built (*l* equals 100 by default). Within each set, the sequences are then compared to find the first divergence between all sequences. A new node is added to the graph, containing the repeated portion of the sequences, and repeated nodes are shortened accordingly. This process is applied iteratively to every node, including the newly created nodes, for which a subset of neighbors may still show identical sequences.

Finally, simple linear paths, with no branching nodes, are merged into a single node.

After the simplification process, the resulting graph may not be a linear sequence because of intrasample polymorphism or assembly uncertainties. Although the genome graph is the main output of MinYS, it may be necessary to analyze it further to get conventional linear assemblies. This can be done either by manual inspection using interactive tools such as Bandage (23) or by enumerating all possible paths within the graph.

#### Implementation and availability

MinYS is implemented as a python3 program and available at https://github.com/cguyomar/MinYS and as a conda package in the Bioconda repository. Notably, it relies on the local assembly tool MindTheGap, starting from version 2.1 that enables the so-called contig mode (https://github.com/GATB/MindTheGap). The whole pipeline presented in Figure 1 can be run in a single command line with few parameters to tune and is implemented in a modular way enabling to start or resume the analysis at intermediary steps. The most influential parameters concern the two *de novo* assembly steps (backbone and gap filling). They are standard parameters of de Bruijn graph-based assemblers that depend mainly on the expected read depth of the targeted genome in the sample, namely the size of *k*-mers and the *k*-mer minimal abundance used to filter out sequencing errors.

### Application to pea aphid metagenomic datasets

In this study, we applied MinYS to the assembly of the pea aphid obligatory symbiont, *B. aphidicola*. We considered 50 pea aphid resequencing samples of Illumina 100-bp paired-end reads (24). Thirty-two samples contain each the sequences from a single aphid clone. In addition, 18 samples sequenced from pooling 14–35 genetically distinct pea aphid individuals were also analyzed. These samples are more challenging for metagenomic assembly due to their richer symbiotic composition and strain diversity (24).

Scripts and commands used to perform all the analyses and create tables and figures are available and documented on the following GitHub repository: https://github.com/cguyomar/MinYS_paper_reproducibility.

#### Reference genomes with increasing levels of divergence

Reference-guided assembly was performed with four distinct reference genomes of *B. aphidicola* with increasing levels of divergence: (i) *B. aphidicola* from *A. pisum* (LSR1 accession), hereafter called *closest*, which is the closest available assembled genome; (ii) *B. aphidicola* strain G002, which was isolated from a different aphid host, *Myzus persicae*, hereafter called *distant*; (iii) *B. aphidicola* strain Sg, isolated from an even more distant aphid host, namely *Schizaphis graminum*, the most divergent reference analyzed and hereafter called *most distant*; and (iv) a synthetic genome obtained by deleting 116.4 kb of sequences from the closest reference genome (*B. aphidicola* LSR1) and referred to as *incomplete*. This synthetic genome was generated by applying 20 deletions, whose size ranged from 300 bp to 20 kb. The levels of divergence of these different reference genomes are supported by phylogenetic studies (25), whole genome alignments [average nucleotide identity (ANI)] and relative amounts of mapped reads from the real sequencing samples, compared to the closest reference genome (see Table 1).

#### Dataset with simulated structural variations

To assess the ability of MinYS to recover structural variations in samples with strain diversity, we created a synthetic pea aphid sample by adding to a real sample a subset of simulated reads from the previously described *incomplete* genome (with 20 deletions). A 50× coverage of 100-bp reads was simulated with wgsim of the SAMtools suite (26), using the parameters ‘-1 100 -2 100 -N 131400’.

#### MinYS parameters

MinYS was used in version 1.1, as available on Bioconda. Two sets of parameters were used to run MinYS, depending on the sample coverage. Since the coverage of pooled samples was greater than that of the individual ones, more stringent settings were chosen. For individual samples (pooled samples), a *k*-mer size of 61 (81) was chosen for the assembly step, along with a *k*-mer minimal abundance threshold of 10 (20) and a minimum contig length of 400 bp. The gap-filling step was performed with a *k* value of 51 (71) and a *k*-mer minimal abundance threshold of 5 (10), and the arguments *max-length* and *max-nodes* were set to 50 000 and 300 (1000).

#### Path enumeration in genome graphs

Genome graphs incorporate the genomic diversity present in a sample and therefore can often not be simply converted in a single genomic sequence. In order to provide standard assembly metrics and to allow comparisons with other assembly approaches, genome graphs were analyzed to recover a single genome sequence when only punctual polymorphism or small assembly uncertainties distinguished the different paths in the graph, or otherwise a set of genome sequences that were representative of the different genome structures. For each connected component of the graph, all possible paths were enumerated. A clustering of those paths was performed in order to output a subset of substantially different sequences. Paths were compared using the ANI metric, as implemented in pyani (27). Two paths were considered identical if an alignment with >99% of sequence coverage and >99% of nucleotide identity could be obtained. After all the paths have been enumerated and compared, the longest resulting sequence was arbitrary chosen and considered as the representative genome sequence, for calculating assembly metrics and making comparisons with other approaches.

#### Comparison with other approaches

Alternative usual approaches to assemble a particular genome from metagenomic data were applied on the 50 pea aphid samples using the *distant* reference genome.

Three reference-guided assembly tools were used: MITObim (13), NOVOPlasty (14) and MetaCompass (18), which is based on Pilon (16). MITObim was run with the ‘-quick’ parameter allowing the user to supply a reference genome for read baiting, and a maximal number of iterations of 31. NOVOPlasty was run using the mitochondrial genome assembly mode and recommended parameters, with a *k*-mer size of 39. MetaCompass was used in its reference-guided mode with default parameters. Alongside reference polishing using Pilon, MetaCompass also uses MEGAHIT (11) to assemble all unmapped reads. As such, the assembly returned by MetaCompass is not targeted and contains contigs representing all the sequenced genomes. In order to extract only the contigs associated with *B. aphidicola*, we performed a BLAST alignment against the chosen reference genome and retained the contigs with at least 50% of their length covered by BLAST hits with *e*-values <10^−5^. The final MetaCompass assembly therefore includes the Pilon-corrected sequences and the BLAST-filtered MEGAHIT contigs.

Alternatively, two assembly-first approaches were used for comparison. A complete *de novo* assembly was performed for each sample using MEGAHIT (11) and *B. aphidicola* contigs were selected by a BLAST alignment against the chosen reference genome, as in the case of MetaCompass (at least 50% of sequence coverage and *e*-value <10^−5^). As in the case of MinYS, we did not include contigs <1 kb, mainly associated with plasmid sequences. Alternatively, Autometa (8), a reference-free binning approach, was also used to bin *Buchnera* contigs from the metagenomic assembly for one particular pool sample.

All tools were run with 8 CPU cores, with the exception of Autometa that was run with 32 cores to achieve a shorter runtime. The quality of each assembly was assessed using QUAST (28) and the closest reference genome (*B*. *aphidicola* str. LSR1 from *A. pisum*). Several assembly metrics were compared such as their length relative to the targeted genome size, the number of contigs and the length of the largest contig. Those metrics were also computed for the backbone assembly performed at the first step of MinYS, mainly as a way to measure the relative contributions of reference-based assembly and gap filling to the final assembly.

## RESULTS

In this study, we applied MinYS to assemble genomes of *B. aphidicola* (∼640 kb), the obligatory bacterial symbiont of the pea aphid. We considered a large set of pea aphid resequencing samples of Illumina 100-bp paired-end reads, which have already been studied in a previous work, in which the microbiota of each aphid sample was detailed (24). Among the 50 datasets, 32 resulted from the sequencing of individual clones and 18 from the sequencing of pools of individuals from the same population. The number of reads is on average 84 (198) million for individual (pool) sequencing datasets, with an average coverage of 628× (3694×) for the *B. aphidicola* genome. In these datasets, >90% of the reads originate from the insect host and are not useful when focusing on symbiont genomes. This dominance of host reads in our metagenomic datasets justifies the choice of a targeted assembly approach, which does not require assembling pea aphid reads.

### Single contig assembly of the targeted genome using increasingly distant reference genomes

MinYS was first applied to the set of 32 resequenced aphid clones using several reference genomes. Assembly statistics are shown in Table 3. Overall, the *B. aphidicola* genome was very well assembled in most cases: for all samples but one, the targeted genome is assembled in one single contig, whose size is comparable to the expected genome size. Overall, the sequence accuracy was satisfying for all the assessed methods and within the range of the expected divergence to the closest reference genome.

**Figure 2.**
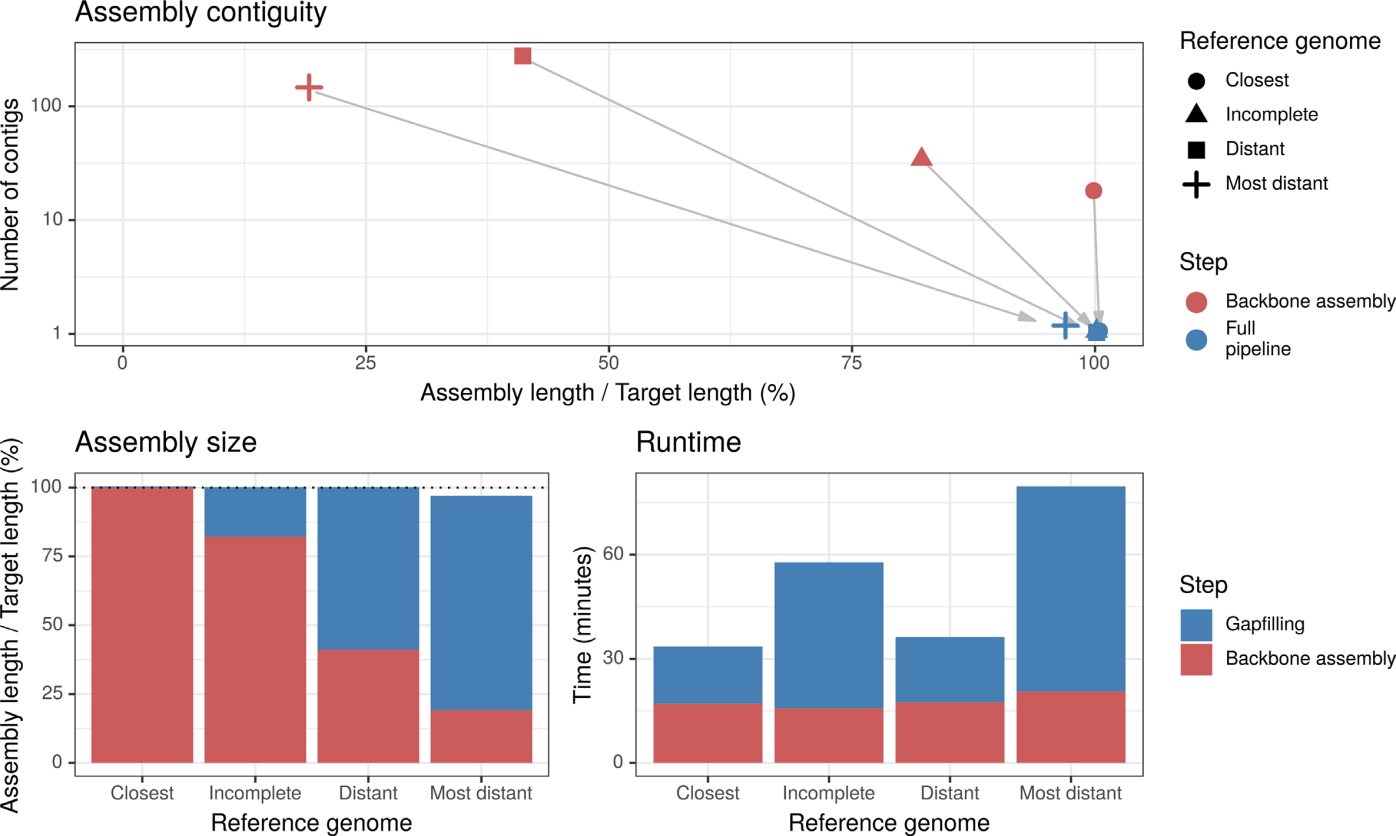
Effect of the level of divergence of the reference genome on MinYS assembly statistics (assembly contiguity in the top plot, assembly completeness in the bottom left plot and running time in the bottom right plot) and on the relative contribution of the first backbone assembly step (in red) and of the gap-filling step (blue). The plots show the average values for the 32 individual samples.

As MinYS relies on a reference genome to perform the assembly, we compared the assembly results using increasingly distant reference genomes (Table 1). Figure 2 presents the results obtained with the different genomes and shows the contributions of the first (reference-based mapping and assembly) and second (gap filling) steps of MinYS. The main result is that using an incomplete or distant genome did not affect the assembly quality: both assembly length and contiguity are similar to those obtained using the closest genome. Using the incomplete genome, which includes 20 deletions between 300 bp and 20 kb, did not impede the assembly since all missing regions from the reference genome were well assembled, highlighting the power of MinYS to recover large previously unknown regions of the genome. Only the use of the most distant genome (*B. aphidicola* from *S. graminum*) decreased the completeness and contiguity of some assemblies. In this extreme case, still 88% of samples were assembled in a single contig, and 90% of the assemblies had their length >98% of the targeted genome length.

**Table 1.** Description of the different reference genomes of *B. aphidicola* used in this study and their levels of divergence with the targeted genome

	Name	Host	Accession	Length (bp)	Proportion of mapped reads	ANI
Closest	*B. aphidicola* str. LSR1	*A*.*pisum*	NC_002528.1	642 011	100%	100%
Incomplete	*B. aphidicola* str. LSR1	*A*.*pisum*		525 611	81%	100%
Distant	*B. aphidicola* str. G002	*M. persicae*	NZ_CP002701.1	643 517	48%	81%
Most distant	*B. aphidicola* str. Sg	*S*.*graminum*	NC_004061.1	641 454	30%	78%

ANI stands for average nucleotide identity and is computed on the aligned portions of the genome.

**Table 2.** Graph complexity metrics of the genome graphs output by MinYS for individual (top) and pooled (bottom) samples with several reference genomes used as a guide

	Reference genome				
	Closest	Incomplete	Distant	Most distant	Average
**Individual samples**					
Assembly in a single connected component	93.75%	93.75%	96.88%	87.50%	92.97%
Assembly in a single path (before comparison)	71.88%	71.88%	81.25%	81.25%	76.57%
Assembly in a single path (after comparison)	96.88%	96.88%	96.88%	93.75%	96.10%
**Pooled samples**					
Assembly in a single connected component	100.00%	100.00%	100.00%	88.89%	97.22%
Assembly in a single path (before comparison)	11.11%	5.56%	5.56%	0.00%	5.56%
Assembly in a single path (after comparison)	55.56%	61.11%	88.89%	27.78%	58.34%

Values indicate the percentage of samples with the given graph characteristics.

However, using more distant reference genomes affected the intermediate results of the MinYS pipeline. Using more distant reference genomes resulted in fewer reads mapped and assembled during the first step of the pipeline, and therefore in a more partial initial backbone assembly. The fraction of the genome assembled during the first mapping and assembly step was 99% for the *closest*, 47% for the *distant* and 24% for the *most distant* reference genome. As a consequence, the gap-filling step is essential when using more distant genomes. Since final assembly results are comparable, this shows that this step successfully recovers the genome portions highly divergent or missing from the reference genome. Accordingly, the running time increases when a remote reference is used, mainly due to the increase of time devoted to gap filling.

### Assembly of pooled samples with a higher strain diversity

MinYS was then applied to an additional set of 18 sequencing samples of pooled aphids (with 15–34 individuals per pool). An important feature of MinYS is to output a genome graph that can represent several putative assemblies. In the case of pooled samples, the genome graph output by MinYS encompasses more diversity than in individual samples, as shown by the higher number of enumerable paths in these graphs (see Table 2). While 77% of the individual samples were assembled in a single path, it was the case for only 6% of the pooled samples. This shows that MinYS is able to recover the higher level of strain diversity present in pooled samples. Interestingly, most alternative paths in these pooled samples differed only by punctual polymorphisms and did not encompass large structural variations. Clustering the enumerated paths for each sample with a 99% sequence identity threshold resulted in one single representative sequence for 58% of the pooled samples. Noteworthy, using the most distant reference genome as a primer resulted in more complex graphs and pushed to its limits this post-processing step of path clustering. In this case, four samples were not considered due to excessively high computational time for the comparison of sequence paths. When more than one representative sequence was generated, the longest path was arbitrary selected, explaining why the assembled length is slightly higher in pooled samples (see Table 3).

### Assembly of coexisting structural variants

**Figure 3.**
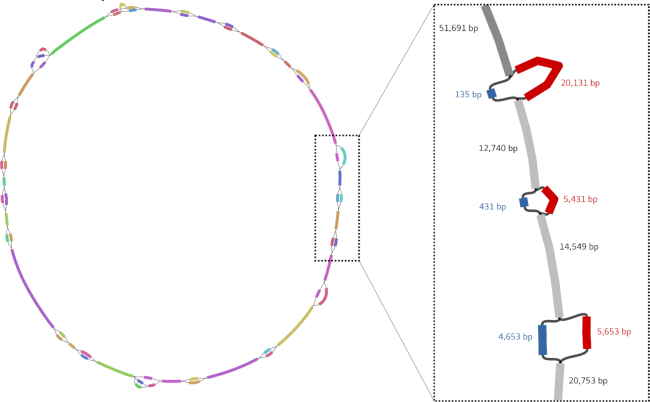
View of the genome graph generated by MinYS with a sample containing two coexisting strains of *B. aphidicola* differing by 20 structural variants. The graphical representation was obtained with the assembly graph visualization tool, Bandage, where each node is represented by a colored rectangle whose size is related to the node sequence size.

MinYS was applied to a pea aphid sample in which simulated reads from a rearranged *B. aphidicola* genome were added to a real pea aphid resequencing sample, simulating the coexistence in a metagenomic dataset of two strains with structural differences (here 20 deletions whose sizes ranged from 300 bp to 20 kb). In the resulting genome graph, 17 out of the 20 simulated deletions were fully recovered, with both the deleted and complete versions of the genome assembled (Figure 3). Extracting the longest path from the graph resulted in a single contig of 641.5 kb, compared to the 642 kb of the closest reference genome. Similarly, the shortest path extracted from the graph was 526.4 kb long, compared to 525.6 kb for the simulated deleted genome. The longest structural variants (up to 20 kb) were all successfully recovered. Only two small variants, of 300 and 500 bp, were missing from the graph.

### Comparison with other approaches for targeted genome assembly

MinYS assemblies were compared to the results of other reference-based approaches [MITObim (13), NOVOPlasty (14), MetaCompass (18)] and a *de novo* metagenomic assembly [MEGAHIT (11)] followed by BLAST-based contig filtering. Results using the *distant* reference genome are shown in Table 3.

**Table 3.** Assembly metrics of different assembly approaches using the *distant* reference genome

Tool	Assembly/target length (%)			No. contigs			Largest contig (kb)			Runtime (h)		
	Indiv.	Pool	Worst	Indiv.	Pool	Worst	Indiv.	Pool	Worst	Indiv.	Pool	Worst
MinYS	100	101	114	1.0	1.0	2	642	646	437	0.64	2.34	4.51
NOVOPlasty	100	100	0	1.0	1.0	9	642	642	0.1	0.66	3.10	8.67
MetaCompass	116	124	138	54.5	90.0	148	642	310	117	6.69	29.16	104.87
MEGAHIT	100	102	104	1.5	11.5	30	642	366	86	5.00	15.98	34.44

For each metric, the median values obtained over all individual (Indiv.) or pooled (Pool) samples are given, as well as the worst value obtained over all the samples (Worst). Extended statistics are available in Supplementary Table S1.

MITObim was designed for reference-guided assembly of mitochondrial genomes by iterative read baiting and mapping (13). MITObim was stopped after 51 h of runtime, reaching eight iterations. It returned an incomplete assembly, with 221 kb missing (33% of the genome). As such, it does not scale up to genomes larger than mitochondrial genomes, and was not considered as an alternative to MinYS in the present work.

NOVOPlasty is another organelle assembly tool that can be used to assemble other circular genomes. Similarly to MinYS, it may output several paths within a contig graph, with the additional constraint that only cyclic graphs can be analyzed. Out of the 50 metagenomic samples, 31 were assembled as a single path. After selecting one representative path for the other samples, overall 44 samples were assembled in a single contig whose size is close to the target genome size of 642 kb (compared to 47 samples for MinYS). For the remaining six samples, the assembly sizes deviated substantially from the target genome size, ranging from 100 bp (no assembly was performed) to 1.06 Mb (see the extended assembly statistics in Supplementary Table S1).

MetaCompass first polishes the supplied reference genome sequence with mapped reads using Pilon (16) and then assembles *de novo* the remaining unmapped reads using MEGAHIT. To recover, among the MEGAHIT contigs, only the ones originating from the genome of interest, an additional step of mapping to the reference genome is therefore necessary and was performed here with BLAST. The first step was insufficient to return satisfying assemblies in this situation where we are using a distant reference genome. Considering only the polished sequences returned by Pilon resulted in far from complete assemblies, with an average length of 6% of the targeted length for individual samples. Therefore, the assemblies described in Table 3 were dominated by *de novo* assembled contigs, mitigating the referenced-based approach of MetaCompass. Overall, obtained assemblies were more fragmented and longer than expected, due to potential redundancy between polished and *de novo* contigs.

Finally, we compared MinYS to a full metagenomic assembly approach with MEGAHIT, followed by BLAST-based contig filtering. On individual clone samples, MEGAHIT performs on par with MinYS, resulting in complete one-contig assemblies in most cases. However, when applied to samples of pooled individuals with more polymorphism, MEGAHIT does not perform as well, with more and shorter contigs (the median size for the largest contig is 57% of the expected genome length, compared to 101% with MinYS). An explanation for this lower performance could be that highly polymorphic regions may be assembled by MEGAHIT into distinct contigs that break the contiguity, while MinYS often represents them as bubbles in the genome graph.

Notably, the metagenomic assembly approach with MEGAHIT performed poorly compared to MinYS on the metagenomic dataset simulating the co-existence of structural variants. It resulted in a 38-contig assembly, with a total length of 646 kb and an N50 of 44.5 kb, whereas several structurally different strains can be extracted from the genome graph output by MinYS in a single contig each. This highlights the difficulty of *de novo* assembly to deal with structural diversity in metagenomic samples.

An alternative approach to select *Buchnera* contigs from the whole MEGAHIT assembly was tested. Autometa is a binning method dedicated to extract microbial genomes from a single sample of a eukaryotic host. It was used on a particular sample for which the BLAST-based filtering performed poorly compared to MinYS: 24 contigs with a total size exceeding the expected size by 20 kb. On this sample, Autometa reduced the number of contigs to 8 but did not reduce the genome size; on the contrary, the latter increased with 34 kb in excess. Importantly, this approach required a substantial increase of running time of 20 h with respect to the BLAST-based filtering, using 32 CPU cores instead of 8 for the other approaches. Assembly and contig binning took overall 40 h, whereas MinYS assembled a single contig genome in 4.5 h for this sample. This large increase in running time led us to leave this strategy behind for the other samples.

Importantly, MinYS was substantially faster than all other tested tools (Table 3). Being designed for smaller organelle genomes, NOVOPlasty struggled to scale up to bacterial genomes. The runtime for pooled samples was 33% greater for NOVOPlasty compared to MinYS, and the median peak memory usage reached 42 GB for individual samples (6.7 GB for MinYS) and 187 GB for pooled samples (9.1 GB for MinYS). The average runtime of MinYS was 38 min for individual samples and 6.5 h for pooled samples, which was, respectively, 10 and 12 times smaller than MEGAHIT runtimes. Indeed, MEGAHIT produces contigs not only for the targeted organism, but in this case for the insect host *A. pisum* and all its secondary symbionts. This highlights the importance of a tool such as MinYS to efficiently recover specific genomes of interest from metagenomic data.

## DISCUSSION AND CONCLUSION

### *De novo* and reference guided assembly: the best of both worlds?

When working on microbial communities, focusing on the genome assembly and characterization of a particular organism can be especially relevant. For biologists, understanding these communities can be achieved by focusing on some key players with particular functional effects or ecological impacts. For bioinformaticians, assembling a single genome can be significantly less challenging than a whole community, especially in the context of symbiotic associations where reads originating from the organism of interest are a minority within host-dominated sequence data. Yet, existing tools for targeted assembly are usually not suited for metagenomic data, or rely on reference sequence correction, which is unable to capture novel sequences absent from the reference genome.

MinYS is a novel method leveraging the benefits of both reference-based and *de novo* assembly. By using a reference genome as a primer for the assembly, MinYS significantly reduces the computational burden of genome assembly from a metagenomic sample. In the first step, the assembly is restricted to a subset of the reads, and is therefore usually straightforward. During gap filling, although the whole readset is used, performing local assembly between specific source and target *k*-mers is also less demanding than complete metagenomic assembly. In addition, as long as trustworthy contigs are assembled in the first place, this local gap filling is less prone to include contaminant sequences. This makes the assembly both more reliable and faster. In this context of mining a particular symbiont genome out of a host-dominated sequencing dataset, MinYS was up to 12 times faster than a full metagenomic assembly.

MinYS obtained also better contiguity statistics, with the genome of *B. aphidicola* being assembled in one circular sequence in most cases. Admittedly, this organism has a small and simple genome containing few repeated sequences, enabling to obtain full-length assemblies with short read data. Although microbial communities associated with a host could include more complex genomes, symbiont genomes have generally small sizes (5), making our approach applicable to many other host–symbiont interactions. Moreover, although apparently simple, our analyses have shown that depending on the sequencing context, the assembly task may not be so straightforward, as highlighted by the lower contiguity of MEGAHIT assemblies when strain diversity was present in the sample. In this context, MinYS showed significant improvements in assembly contiguity with respect to other approaches. Although full metagenomic assembly also delivers contigs for the other organisms of the community, we consider that the speed increase along with the contiguity improvement makes MinYS a worthy alternative to analyze particular components of microbial communities.

Dedicated host–symbiont contig binning approaches, such as BlobTools and Autometa, rely heavily on the quality of the initial full metagenomic assembly. Bypassing this full assembly also makes MinYS a more generalizable approach to a variety of situations where *de novo* assembly is difficult, such as low-coverage host genome resequencing, pooled sequencing or more complex metagenomic datasets.

### MinYS performs reference-based assembly, but not reference-dependent assembly

Alternative reference-based approaches usually rely on read mapping followed by a correction of the existing reference genome. On the contrary, every sequence returned by MinYS has been assembled from the metagenomic reads. This reduces the bias due to the choice of a reference genome. Thanks to the gap-filling step, it is also possible to assemble *de novo* regions too distant or absent in the reference genome to be captured by reference-based approaches. These regions represented up to 80% of the targeted genome in the most extreme case considered here.

Remarkably, MinYS proved to be particularly robust with respect to the level of divergence with the chosen reference genome. Thanks to its two-step approach, MinYS can adapt to different divergence levels. When using a close reference genome, the first mapping-based step is predominant, and the assembly is fast. On the contrary, when using more distant reference genomes, the gap-filling step is essential to recover regions missing or too divergent compared to the reference. MinYS can deal with both a high level of divergence and the presence of large novel inserted sequences. When based on an incomplete reference genome, it was able to recover all the missing sequences (including up to 20 kb contiguous missing parts).

In this work, a single set of parameters was chosen for all samples, regardless of divergence or sample coverage. However, MinYS is flexible and also allows more or less conservative parameters to be used to adapt to particular use cases, for instance to accommodate with higher levels of divergence. Overall, although MinYS relies on a reference genome as a primer for the assembly, its hybrid approach enables the use of distant reference genomes, while still being able to return full-length assemblies.

### MinYS assembles and allows visualization of genomic diversity

Species in metagenomic samples may not be homogeneous populations sharing the same genome. As highlighted by the results of MEGAHIT on pooled samples, a high level of polymorphism within a sample affects the contiguity of the assembly. Genomic *loci* showing significant nucleotide diversity are assembled in separate contigs instead of single consensus contigs and they fragment the assembly. In MinYS, such *loci* are deliberately represented as distinct nodes in the genome graph, but are still connected together. As such, the genome graph encompasses different assemblies and its complexity reflects the genomic diversity in the sample. Accordingly, pool sequencing samples, which include a greater microbial diversity (24), were assembled into more complex graphs. These graphs contain biologically meaningful information that surpasses the ‘one species–one genome’ paradigm, which has been strongly criticized (29,30). As a consequence, many novel tools are being developed to use such genome graphs as a reference rather than a conventional linear sequence, for instance for read mapping or variant genotyping (31,32).

Genome graph representation is also a powerful way to represent structural variations in metagenomic samples. In the context of two coexisting strains differing by large structural variants, MinYS was able to reconstruct both strains as alternative paths in the genome graph, while MEGAHIT yielded a fragmented assembly.

Genome graphs offer the opportunity to better represent and explore the genomic diversity in metagenomic samples, but they are also challenging objects to study. The main output of MinYS is a genome graph that may require some post-processing to output one or several representative sequences. Here, all possible paths in the graphs were enumerated and compared to each other to choose a single representative genome. Additionally to the computational burden of such an enumeration, many of these paths may not represent actual strains. Therefore, an interesting but far from trivial perspective of this work would be to map back the paired-end reads on the graph in an attempt to phase the different variants, and then to provide a more accurate representation of the genomes of the strains actually present in the sample. Furthermore, long-range information provided by long-read or linked-read sequencing technologies could help not only to disentangle the different strains at play but also to simplify the genome graph for genomes containing many repeated sequences. As a matter of fact, several software exist to map noisy long reads to genome graphs in the GFA format, such as GraphAligner (32) and vg-map from the vg toolkit (31). Thanks to its standard GFA format output, it would be relatively straightforward to pipeline such tools to the MinYS output. As the high throughput of short-read technologies remains an important asset for studying strain diversity within microbial communities, we anticipate that MinYS will remain a valuable tool that evolves over the advance of sequencing technologies.

## Supplementary Material

lqaa047_Supplemental_File
