# Supplementary material for: MinYS: mine your symbiont by targeted genome assembly in symbiotic communities
Source: NAR Genom Bioinform. 2020 Jul 3;2(3):lqaa047. doi: 10.1093/nargab/lqaa047 (PMC7671366; doi:10.1093/nargab/lqaa047)
Supplement: lqaa047_Supplemental_File [file lqaa047_supplemental_file.pdf]

Supplementary material for MinYS: Mine Your Symbiont by targeted genome assembly in symbiotic communities

Cervin Guyomar, Wesley Delage, Fabrice Legeai, Christophe Mougel, Jean-Christophe Simon and Claire Lemaitre

1 Contig mode of MindTheGap

The gap-filling step of MinYS is based on a module of the software *MindTheGap*, originally developed for the detection and assembly of long insertion variants [1]. The original *fill* module of *MindTheGap* takes as input a read set and a set of insertion variant breakpoints, of the form of two kmers, the left and right kmers adjacent to each insertion site. It first builds a de Bruijn graph of the entire input read set, and then performs a local assembly between the left and the right kmers of each insertion site, by looking for all the paths in the De Bruijn graph starting from the left (source) kmer and ending in the right (target) kmer.

In this work, we took advantage of this module of *MindTheGap* and adapted it to the problem of simultaneous gap-filling between multiple contigs. It has been modified to make possible the gap-filling between a source kmer and **multiple** target kmers, enabling the "all versus all" gap-filling within a set of contigs with only a linear increase of the runtime (compared to a quadratic increase for a naive "all versus all" gap-filling as if using *MindTheGap* in its original breakpoint mode).

The resulting algorithm is illustrated in Figure S1. Two *source* kmers are extracted for each contig: at the end of it and at the end of its reverse complement sequence, resulting in a set of $2n$ *source* kmers for n contigs. Similarly, a set of $2n$ *target* kmers is extracted at the beginning of all contigs and their reverse-complements. For each source kmer, a contig graph is created by starting from the source kmer and performing a breadth-first traversal of the *De Bruijn* graph built from the whole readset. Contigs are consensus sequences returned by removing graph motifs such as bubbles (SNPs) and tip-ends (errors). In the contig graph, contigs are nodes, and edges represent the existence of a $k-1$-mer overlap between two contigs. The algorithm creation of the contig graph is similar to the one used in the *Minia* assembler [2]. The traversal is stopped when the graph becomes too large (total assembled nucleotides) or too complex (number of contigs), according to user-defined parameters. Importantly, if one of the target kmers is found during the contig graph

construction, that contig is not extended further, avoiding redundant contig assembly, and saving time and memory. After the contig graph has been built, target kmers are searched within this contig graph, and gap-filling sequences are built, by traversing the contig graph from each target kmer to the source kmer. For each source-target couple, if several sequence solutions are returned, redundant solutions above a 95% identity threshold are removed. Thanks to this multi-target version of the algorithm, only $2n$ contig graph constructions are necessary to search all possible gap-filling sequences between all pairs of contigs in both directions, instead of n^2 with the naive approach.

The whole process is parallelized by dispatching the $2n$ starting kmers to different threads. The main output is a genome graph in the GFA format (Graphical Fragment Assembly, `https://github.com/GFA-spec/GFA-spec`), containing all input contigs and their gap-filling sequences as nodes, together with their overlap relationships as edges.

All these changes have been implemented in the so-called *contig mode* of *MindTheGap* (since version 2.1), whose input is a read set together with a set of contigs and whose output is a sequence graph in GFA format. *MindTheGap* is freely available at `https://github.com/GATB/MindTheGap`.

Figure S1: **Gap-filling a set of contigs using *MindTheGap* fill module.**
a) Source and target kmers are extracted from the 3 input contigs, resulting in 2 sets of 6 kmers, source (red) and target (blue) ones. b) A graph of contigs is built starting from the right source kmer of contig A. The extension is stopped when a target kmer of another contig is encountered or a maximum assembly size is reached. c) This results in 3 gap-filling sequences starting from contig A right source, 2 gap-filling sequences joining contig B, and one contig C.

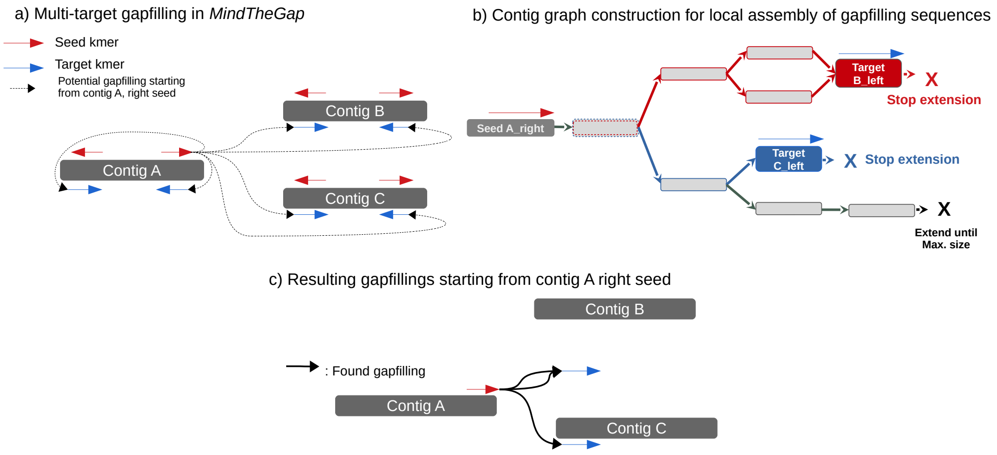

2 Graph simplification

Figure S2: **Graph simplification applied to the gap-filling step output**.
a) Reciprocal gap-fillings with more than 95% identity are merged. b) Shared sequences between gap-fillings originating from or leading to a same contig are merged to reduce sequence redundancy. c) Simple linear paths, with no branching nodes, are merged into a single node.

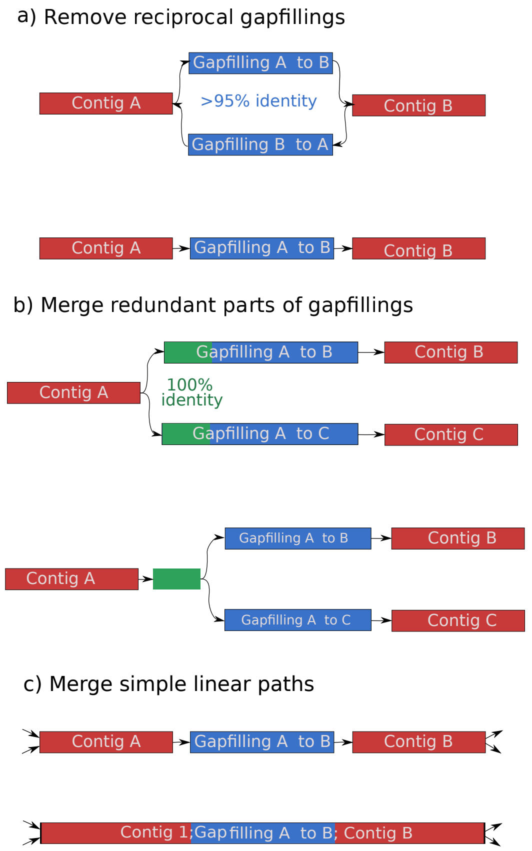

References

[1] G. Rizk, A. Gouin, R. Chikhi, and C. Lemaitre, "MindTheGap: integrated detection and assembly of short and long insertions," *Bioinformatics*, vol. 30, pp. 3451–3457, dec 2014.

[2] R. Chikhi and G. Rizk, "Space-efficient and exact de Bruijn graph representation based on a bloom filter," *Lecture Notes in Computer Science (including subseries Lecture Notes in Artificial Intelligence and Lecture Notes in Bioinformatics)*, vol. 7534 LNBI, pp. 236–248, sep 2012.

3 Full assembly statistics

Table 1: Assembly results obtained by several assembly approaches, using four reference genomes with increasing levels of divergence, for the 50 pooled and individual samples. Values are : median (minimum-maximum).

	Length / reference length (%)		Nb. contigs		Largest contig (kb)		Runtime (h)	
Tool	Individuals	Pools	Individuals	Pools	Individuals	Pools	Individuals	Pools
**Closest**								
MinYS	100 (100-113)	101 (100-109)	1 (1-2)	1 (1-1)	642k (437k-727k)	648k (642k-703k)	0.55 (0.18-1.2)	2.29 (1.33-5.42)
Megahit	100 (100-102)	102 (101-104)	1 (1-9)	15 (5-30)	642k (348k-643k)	366k (86k-642k)	5 (2.14-10.76)	15.98 (2.97-34.44)
**Incomplete**								
MinYS	100 (100-101)	101 (100-107)	1 (1-2)	1 (1-1)	642k (437k-646k)	646k (642k-686k)	0.93 (0.22-1.83)	3.32 (1.26-8.23)
Megahit	100 (100-100)	100 (90-101)	1 (1-3)	4.5 (1-16)	642k (348k-643k)	366k (86k-642k)	5 (2.14-10.76)	15.98 (2.97-34.44)
**Distant**								
MinYS	100 (95-114)	101 (100-109)	1 (1-2)	1 (1-1)	642k (437k-730k)	646k (642k-698k)	0.64 (0.19-0.99)	2.34 (1.27-4.51)
NOVOPlast	100 (0-166)	100 (41-100)	1 (1-9)	1 (1-2)	642k (100-643k)	642k (212k-642k)	0.66 (0.31-1.68)	3.1 (2.12-8.67)
Metacompass	116 (111-121)	124 (121-138)	54.5 (38-77)	90 (73-148)	642k (144k-642k)	310k (117k-642k)	6.69 (2.79-14.78)	29.16 (17.31-104.87)
Megahit	100 (100-102)	102 (101-104)	1.5 (1-7)	11.5 (5-30)	642k (348k-643k)	366k (86k-642k)	5 (2.14-10.76)	15.98 (2.97-34.44)
**Most distant**								
MinYS	100 (78-114)	101 (57-109)	1 (1-3)	1 (1-3)	642k (262k-730k)	646k (265k-698k)	0.84 (-Inf-3.54)	2.66 (1.57-8.18)
Megahit	100 (100-102)	102 (101-104)	1.5 (1-8)	13 (5-31)	642k (348k-643k)	366k (86k-642k)	5 (2.14-10.76)	15.98 (2.97-34.44)
